# 

**Published:** 1844-01

**Authors:** 


### CONTENTS OF No. XXXIII.

OF THE

### British and Foreign Medical Review.

JANUARY, 1844.

---

### PART FIRST.—Analytical and Critical Reviews.

PAGE

ART. I.—1. A Treatise on Food and Diet, with Observations on the Dietetical Regimen suited for Disordered States of the Digestive Organs; and an Account of the Dietaries of some of the principal Metropolitan and other Establishments for Paupers, Lunatics, Criminals, Children, the Sick, &c. By JONATHAN PEREIRA, M.D. F.R.S. & L.S. . . . 1

2. A Treatise on Diet, comprising the Natural History, Properties, Composition, Adulterations, and Uses of the Vegetables, Animals, Fishes, &c. used as Food. By WILLIAM DAVIDSON, M.D. M.R.C.S.E. . . . ib.

3. Food, and its Influence on Health and Disease, or an Account of the Effects of different kinds of Aliment on the Human Body; with Dietetic Rules for the Preservation of the Health. By MATTHEW TRUMAN, M.D. . . ib.

I. Of Foods . . . . . . . 2
1. Elements of Food . . . . . . ib.
2. Alimentary Principles . . . . . 7
3. Compound Aliments . . . . . 15

II. Of Diet . . . . . . . 30
1. Of the Digestibility of Food . . . . . 31
2. Of the Nutritive Qualities of Food . . . . 38
3. Rules of Diet . . . . . . 39
4. Quantity of Food . . . . . . ib.
5. Age . . . . . . . 42
6. Climate and Season . . . . . 44
7. Times of Eating . . . . . . 45
8. Habits in reference to Meals . . . . 46
9. Dietaries . . . . . . 47
10. On the Dietetical Regimen suited for Disordered States of the Digestive Organs . . . . . . 51

ART. II.—A Practical Treatise on the Diseases of the Testis, and of the Spermatic Cord and Scrotum. By T. B. CURLING . . . 55

ART. III.—Om Sygepleien i Straffeanstalterne i Norge. Ved Prof. F. HOLST . 88

On the Management of the Sick in the Prisons of Norway. By Prof. FRED. HOLST.

§

4294

PAGE

ART. IV.—The Transactions of the Provincial Medical and Surgical Association. VOL. XI . . . . . . . 92

3. Mr. ADDISON'S experimental and practical Researches on the Structure and Function of Blood-corpuscles in Inflammation, and on the origin and nature of Tubercles of the Lungs . . . . ib.

4. Dr. WALLIS on the Long Issue in the Scalp . . . 96

5. Mr. CROWFOOT on a case of Fracture of the Spine, treated successfully by Extension . . . . . . . 98

6. Mr. JOHN BANNER on a case of Paralysis of the Serratus Magnus, which caused the lower angles of the right Scapulæ to become disengaged from the Latissimus Dorsi . . . . 99

ART. V.—Die Krankheiten des höheren Alters und ihre Heilung dargestellt von Dr. C. CANSTATT, &c. . . . . . . 100

On the Diseases of Old Age, and their Cure. By Dr. C. CANSTATT, Royal Bavarian Medical Jurist, &c.

ART. VI.—Lectures on the Principles and Practice of Physic, delivered at King's College, London. By THOMAS WATSON, M.D., Physician to the Middlesex Hospital . . . . . . . 119

ART. VII.—Essai d'Hématologie pathologique. Par G. ANDRAL, Professeur de Pathologie, &c. à la Faculté de Médecine, &c. . . . 136

Essay on the Pathology of the Blood. By G. ANDRAL, M.D. &c.

ART. VIII.—Views upon the Statics of the Human Chest, Animal Heat, and Determination of Blood to the Head. By JULIUS JEFFREYS, F.R.S. &c. . 152

ART. IX.—Traité Pratique de l'Amaurose ou Goutte-Sereine, avec des Recherches Nouvelles sur les Méthodes spéciales de Traitement qui conviennent à ses different Espèces. Par J. E. PÉTREQUIN, D.M.P. . . . 155

A Practical Treatise on Amaurosis, containing new Researches on the particular kinds of Treatment adapted to its various Species. By J. E. PÉTREQUIN.

ART. X.—1. On the True Spinal Marrow, and its Anatomy, Physiology, Pathology, and Therapeutics. By MARSHALL HALL, M.D. F.R.S. L. & E. . . 171

2. On the Structure, Relations, and Development of the Nervous and Circulatory Systems, and on the Existence of a complete Circulation of the Blood in Vessels, in Myriapoda, and Macrourous Arachnida. First Series. By GEORGE NEWPORT, Esq., President of the Entomological Society of London, and Member of the Royal College of Surgeons, and Corresponding Member of the Philomathic Society of Paris. From the Second Part of the Philosophical Transactions for 1843 . . . . . ib.

ART. XI.—An Introductory Discourse on the Duties and Conduct of Medical Students and Practitioners. Addressed to the Students of the Medical School of St. George's Hospital, October 2, 1843. By Sir B. C. BRODIE, Bart. F.R.S. &c. &c. . . . . . . 188

ART. XII.—Recherches sur les Echinocoques, chez l'Homme et chez les Animaux. Par EUGÈNE LIVOIS, D.M.P. &c. . . . . . 194

Researches on the Echinococcus in Man and other Animals. By EUGÈNE LIVOIS, D.M.P. &c.

ART. XIII.—Essays on the Determination of Blood to the Head. By ROBERT HULL, M.D., Physician to the Norfolk and Norwich Hospital. . . 200

PAGE

ART. XIV.—1. On the Anatomy and Diseases of the Urinary and Sexual Organs; containing the Anatomy of the Bladder and Urethra, and the Treatment of the Obstructions to which these passages are liable. By G. J. GUTHRIE, F.R.S. &c. &c. . . . . . . 205

2. Traité Pratique sur les Maladies des Organes Génito-urinaires. Par la Docteur CIVIALE. Première Partie: Maladies de l'Urètre. . . . ib.

Practical Treatise on the Genito-urinary Organs. By Dr. CIVIALE. Part First: Diseases of the Urethra.

ART. XV.—1. Some Account of the Epidemic of Scarlatina which prevailed in Dublin from 1834 to 1842 inclusive, with Observations. By HENRY KENNEDY, A.B. M.B. T.C.D. &c., one of the Medical Officers of St. Thomas's Dispensary . . . . . . . . 216

2. Scarlatina, and its Treatment on Homœopathic Principles. By Jos. BELLUOMINI, M.D. . . . . . . . ib.

3. Untersuchungen und Erfahrungen über das Kohlensaure Ammonium und seine Heilkräfte gegen das Scharlachfieber, &c. Von A. W. BODENIUS . ib.

Experimental Researches into the Powers of the Carbonate of Ammonia as a Therapeutical Agent in the Treatment of Scarlatina. By Dr. A. W. BODENIUS.

ART. XVI.—Familiar Letters on Chemistry, and its relation to Commerce, Physiology, and Agriculture. By JUSTUS LIEBIG, M.D. PH.D. F.R.S. Professor of Chemistry in the University of Giessen. Edited by JOHN GARDNER, M.D., Member of the Chemical Society . . . . 224

ART. XVII.—1. A Manual of Medical Jurisprudence. By ALFRED S. TAYLOR, Lecturer on Medical Jurisprudence and Chemistry at Guy's Hospital. . 228

2. Principles of Forensic Medicine. By W. A. GUY, M.B. Cantab., Professor of Forensic Medicine at King's College, London . . . ib.

ART. XVIII.—The Oculist's Vade-mecum: a complete Practical System of Ophthalmic Surgery. By JOHN WALKER . . . . 233

ART. XIX.—Cases of Dropsical Ovaria, removed by the large abdominal Section. By D. HENRY WALNE . . . . . 237

---

### PART SECOND.—Bibliographical Notices.

ART. I.—Animal Physiology. (From the Popular Cyclopædia of Natural Science.) By W. B. CARPENTER, M.D. &c. . . . . 241

ART. II.—Some Observations on the Mental State of the Blind and Deaf and Dumb; suggested by the case of Jane Sullivan, both blind, deaf, dumb and uneducated. By R. FOWLER, M.D. F.R.S. . . . . 242

ART. III.—General Therapeutics and Materia Medica, adapted for a Medical Textbook. By ROBERT DUNGLISON, M.D., Professor of Institutes of Medicine in Jefferson Medical College, Philadelphia, &c. . . . ib.

PAGE

ART. IV.—Elementary Instruction in Chemical Analysis. By Dr. C. REMIGIUS FRESENIUS, Chemical Assistant in the Laboratory of the University of Giessen. Translated by J. LLOYD BULLOCK, Member of the Chemical Society, &c. . . . . . . 244

ART. V.—On Man's Power over himself to prevent or control Insanity. By the Rev. JOHN BARLOW, M.A. F.R.S. &c., Secretary to the Royal Institution of Great Britain . . . . . . 245

ART. VI.—Remarks on Schools of Instruction for Military and Naval Surgeons, in a Letter to Sir Robert Peel. By Sir GEORGE BALLINGHALL, M.D., Regius Professor of Military Surgery in the University of Edinburgh . . 246

ART. VII.—The Electro-physiology of Man. With practical illustrations of new and efficient modes of Galvanic Treatment in a variety of cases. By JOHN DODDRIDGE HUMPHREYS, Esq. . . . . . ib.

ART. VIII.—A Pathological and Physiological Essay on Hereditary Diseases. With an Appendix on Intermarriage, and on the Inheritance of the Tendency to Moral Depravities and Crimes. By JULIUS HENRY STEINAU, M.D. of the Royal Medical College, Berlin . . . . 247

ART. IX.—Manual of British Botany, containing the Flowering Plants and Ferns, arranged according to the Natural Orders. By CHARLES C. BABINGTON, M.A. F.L.S. F.G.S. &C. . . . . . 248

### PART THIRD.—Original Reports and Memoirs.

Report on the Progress of Anatomy and Physiology in the Year 1842-3. By JAMES PAGET, Lecturer on General and Morbid Anatomy and Physiology, and Warden of the Collegiate Establishment at St. Bartholomew's Hospital. 249

Blood . . . . . . . ib.
Fibro-cellular Tissue . . . . . . 251
Muscular Tissue . . . . . . 252
Circulation . . . . . . 253
Respiration . . . . . . 254
Digestion . . . . . . . 257
Absorption . . . . . . . 259
Transformations of Nutritious Substances . . . . 260
Organs of Animal Life . . . . . 261
Nervous System . . . . . . 263
Special Senses . . . . . . 269
Generation . . . . . . . 270
Lactation . . . . . . . 274
Physical History of Man . . . . . ib.

### PART FOURTH.—Medical Intelligence.

1. New Charter of the College of Surgeons . . . . 279
2. Chronological Schedule of the Royal College of Surgeons . 283
3. Naval Lunatics at Haslar . . . . . 285
4. Calculous Diseases in the Mauritius . . . . 288

OBITUARY—Life and Character of THOMAS HARRISON BURDER, M.D. . 289

BOOKS RECEIVED FOR REVIEW . . . . . 292

